# Coronaviruses in the Sea

**DOI:** 10.3389/fmicb.2020.01795

**Published:** 2020-07-24

**Authors:** Gideon J. Mordecai, Ian Hewson

**Affiliations:** ^1^Department of Earth, Ocean and Atmospheric Sciences, The University of British Columbia, Vancouver, BC, Canada; ^2^Department of Microbiology, Cornell University, Ithaca, NY, United States

**Keywords:** coronavirus, Nidovirales, virome, virioplankton, disease

## Abstract

Interest in coronaviruses because of the 2019 novel coronavirus (SARS-CoV-2) pandemic has generated concern about their occurrence and persistence in aquatic habitats. Coronaviruses are not quantitatively significant constituents of marine virioplankton. Members of the *Nidovirales* (to which human coronaviruses belong) infect marine mammals, teleosts and possibly invertebrates, and human coronaviruses may persist in marine plankton receiving wastewater effluent. However, virions likely experience significant particle and infectivity decay rates in surface seawater, similar to other enveloped RNA viruses.

## Introduction

The current 2019 novel coronavirus (SARS-CoV-2) pandemic has generated interest and concern about the occurrence and persistence of coronaviruses in aquatic habitats. While the distal origin of SARS-CoV-2 is still undetermined, but likely terrestrial (bats or other terrestrial animals; Andersen et al., 2020) coronaviruses also occur in aquatic mammals and other metazoa as pathogens. Given reports that SARS-CoV-2 can be shed from patients in feces for prolonged periods (Gu et al., 2020; Hindson, 2020; Wu et al., 2020; Yeo et al., 2020; Zhang et al., 2020) and like other human coronaviruses (Bibby and Peccia, 2013) can be detected in wastewater management facilities (Lodder and De Roda Husman, 2020) it is possible that SARS-CoV-2 may be introduced to aquatic habitats through sewage outfall and contact with infected recreational users. The purpose of this mini-review is to summarize currently known marine (and more generally aquatic) coronaviruses, their presence in plankton, and their potential persistence in aquatic habitats.

## Marine Coronavirus Diversity

Coronaviruses belong to the order *Nidovirales*, a group of viruses rapidly expanding in number mainly as a result of a surge in metatranscriptomic sequencing studies (Shi et al., 2016). Coronavirus virions are large (120–160 nm), and characterized by club-shaped projections on their surface. They bear a ssRNA genome of 25–32 kb, on which (from 5′ to 3′) there are typically two open reading frames encoding non-structural genes, followed by structural genes. Replication of coronaviruses occurs by receptor mediated endocytosis, followed by cytoplasmic replication and assembly of mature virions at the endoplastic reticulum surface, and release by exocytosis (Brian and Baric, 2005). The *Nidovirales* is currently made up of eight suborders (*Abnidovirineae*, *Arnidovirineae*, *Cornidovirineae*, *Mesnidovirineae*, *Monidovirineae*, *Nanidovirineae*, *Ronidovirineae* and *Tornidovirineae*) (King et al., 2018) and virus classification is verified by concatenation and phylogenetic analysis of five protein encoding domains. SARS-CoV-2 belongs to the family *Coronaviridae*, subfamily *Orthocoronavirinae* and genus *Betacoronavirus.*

**FIGURE 1:**
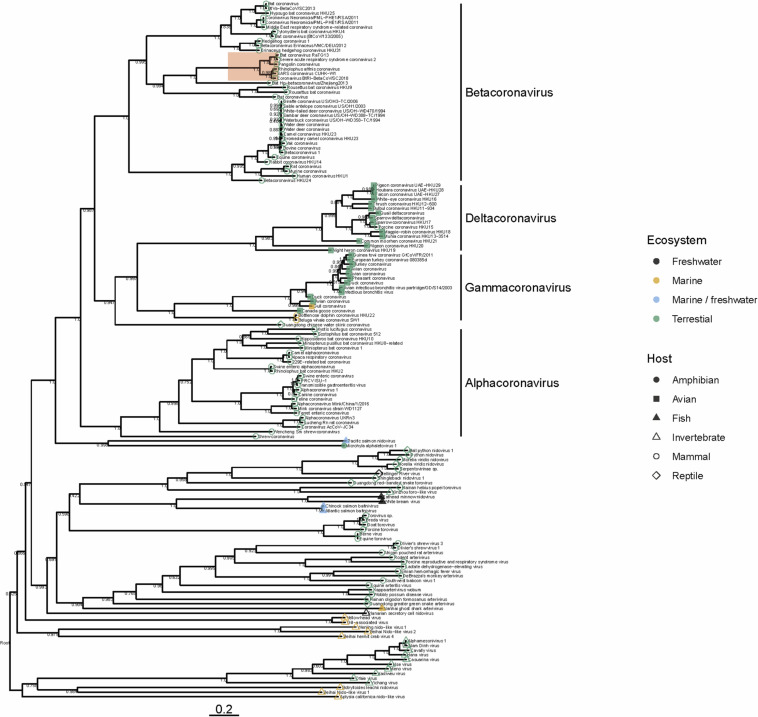
Phylogeny of representative *Nidovirales* based on ORF1a (replicase) amino acid sequences. Closely related sequences were removed. Tip points are colored by ecosystem and shaped by host. The clade highlighted in red shows the phylogenetic placement of SARS-CoV-2 and closely related viruses. Branches are scaled to the number of amino acid substitutions per site and the tree is mid-point rooted. Node values show FastTree support values (ranging from 0 to 1). Amino acid alignments were carried out using MAFFT (Katoh and Standley, 2013) the tree was built using FastTree (Price et al., 2010) and visualized using ggtree (Yu et al., 2017).

To date there have been no Betacoronaviruses recovered from any marine animal. However, Alphacoronaviruses and Gammacoronaviruses are described in marine mammals (reviewed in Schütze, 2016; Bossart and Duignan, 2018). These include the Harbor Seal Alphacoronavirus (Bossart and Schwartz, 1990), Pacific Harbor Seal Gammacoronavirus (Nollens et al., 2010), Beluga Whale Gammacoronavirus (Mihindukulasuriya et al., 2008) and the Bottlenose Dolphin Gammacoronavirus (Woo et al., 2014). These viruses are distantly related to SARS-CoV-2. Gammacoronaviruses and Alphacoronaviruses share little homology with SARS-CoV-2 (Figure 1) but are associated with respiratory diseases in pinnipeds and cetaceans. Gammacoronaviruses and Alphacoronaviruses are associated with respiratory secretions and possibly associated with pneumonia in seals (Nollens et al., 2010; Ng et al., 2011) and respiratory disease in cetaceans (Mihindukulasuriya et al., 2008) however, firm pathology has not been established. Wild birds are known viral reservoirs, and birds which live mainly in the marine environment are also known to harbor coronaviruses. For instance, a novel coronavirus within the Gammacoronaviruses was identified in American herring and great black backed gulls (Canuti et al., 2019). Interestingly, these sea bird coronaviruses are within the same clade as marine mammal coronaviruses (Figure 1), suggesting that in the past, transmission between these animals has occurred. Additionally, and perhaps suggesting that the diversity in the marine environment is not yet fully understood, a Nidovirus (PsNV) recovered from Pacific Salmon shares greater similarity with coronaviruses than to other fish or invertebrate nidoviruses. Interestingly, PsNV was localized via *in situ* hybridization to gill tissue, suggesting a similar tissue tropism and infection strategy to other respiratory coronaviruses (Mordecai et al., 2019, 2020). The morphology, genome organization, and replication of marine gammacoronaviruses is highly similar to human coronaviruses (Mihindukulasuriya et al., 2008; Woo et al., 2014). While no marine gammacoronavirus has been cultivated, the avian gammacoronavirus Infectious Bronchitis Virus (IBV) follows similar entry, replication and shedding as human coronaviruses. While SARS-CoV-2 bind to angiotensin converting enzyme 2 (ACE2) (Yan et al., 2020) IBV binds to sialic acid, and while both infect primarily respiratory tissues, they may have wide tropism and infect multiple organ systems (Winter et al., 2006; Promkuntod et al., 2014; Bande et al., 2016).

Several nido-like viruses have been detected in invertebrates in the marine environment (Shi et al., 2016; Bukhari et al., 2018). Screening of RNA-specific viral metagenomes prepared from asteroids (NCBI Accessions PRJNA253121 and SAMN08012637 – SAMN08012651; Hewson et al., 2014, 2018b) yielded no definitive coronavirus-like sequences, though weak matches based on amino acid homology were found (Hewson et al., 2018b). Analysis of representative Nidovirales diversity shows that the majority of viral discovery studies are focused on terrestrial mammalian and avian hosts, but the few aquatic representatives in the Nidovirales are represented across the tree (Figure 1), suggesting that their absence might be due to inadequate sampling, rather than a restricted host range. It is becoming clear that despite many newly discovered viruses remain unclassified, members of the family *Coronaviridae* are no longer limited to viruses of birds and mammals, but also invertebrates (Shi et al., 2018) reptiles (Shi et al., 2018) amphibia (Bukhari et al., 2018) and perhaps also fish (Mordecai et al., 2019; Figure 1). Our analysis suggests that nidoviruses are likely found in all animals, and that much broader sampling and metatranscriptomic sequencing of marine organisms is required to more fully understand the diversity of viruses found in this environment.

To assess the occurrence of coronaviruses as free virions in plankton (i.e., virioplankton), we screened plankton (>0.2 μm) metatranscriptomes of the New York Finger Lakes (NCBI accessions SRR6281416 – SRR6281423), viral metagenomes the Anacostia River in Washington DC (NCBI accession PRJNA637530), along with published RNA viromes from marine and freshwater virioplankton (Culley et al., 2003, 2006, 2014; Djikeng et al., 2009; Lang et al., 2009; Lopez-Bueno et al., 2015; Hewson et al., 2018a) by BLAST, but these yielded no definitive Orthocoronavirus-like sequences. Zeigler et al. (2017) reported coronaviruses-like sequences in plankton recovered from the Baltic Sea through combined metatranscriptome and virome sampling, but concluded these were likely a result of contamination from human sources. While these libraries represent a tiny fraction of the total diversity of aquatic habitats and aquatic animal diversity, it can be inferred that coronaviruses do not represent numerically significant constituents of virioplankton communities.

## Human Coronaviruses in Marine Ecosystems

As SARS-CoV-2 is likely to be released to the marine environment via human effluent, it is important to understand the impact this might have on marine life (if any). Wastewater surveillance strategies to detect SARS-CoV-2 are currently formulated to track human epidemiology, since these precede case loads in the human population (Daughton, 2020; Randazzo et al., 2020). Given the detection of SARS-CoV-2 nucleic acids in wastewater (Ahmed et al., 2020; Lodder and De Roda Husman, 2020; Medema et al., 2020; Orive et al., 2020) coronaviruses may be introduced into aquatic habitats through urban or agricultural runoff or in wastewater effluent. Indeed, emerging reports at the time of publication indicate the presence of SARS-CoV-2 in river water receiving untreated human sewage (Guerrero-Latorre et al., 2020; Haramoto et al., 2020; Rimoldi et al., 2020). Human coronaviruses (HCoV) experience a 99% loss in infectivity in primary and secondary treated wastewater effluent after 1.9–2.4 days (Gundy et al., 2009). A study by Ye et al. (2016) found that murine hepatitis virus (MHV; coronavirus) experienced 90% reduction in infectivity after 13 h in raw wastewater at 25^*o*^C, but infectivity was maintained longer (36 h) at 10^*o*^C. Taken together, these studies illustrate that intact coronavirus particles may survive in wastewater after excretion, and may be present in coastal waters after discharge. However, coronavirus particles are likely to experience considerable particle decay and loss of infectivity after arriving in aquatic habitats. Virus-like particles, which include both viruses of eukaryotes and phage, experience particle decay in seawater at rates of 2–4 % h^–1^ (Heldal and Bratbak, 1991) which is generally considered to be a result of sunlight (UV-C radiation) (Wommack et al., 1996; Wilhelm et al., 1998) and through interaction with heat-labile organic matter which may include nucleases and proteases present in marine microorganisms and free in the environment (Noble and Fuhrman, 1997). Different viral groups may have different particle decay rates (Wilhelm et al., 1998) and decay of virus particles is a different process to loss of viral infectivity (Wommack et al., 1996). The decay of coronaviruses in natural waters has not been studied. Several fish pathogens, including viral haemorrhagic septicemia virus (VHSV; Rhabdovirus), infectious salmon anemia virus (ISAV; Orthomyxovirus), Salmon Alphavirus (SA; Togavirus), and Infectious Hematopoeitic Necrosis Virus (IHNV: Rhabdovirus) are enveloped RNA viruses (Crane and Hyatt, 2011) which may be detected in fish pens or holding tanks (Pinto et al., 1993) and natural waters (Lovdal and Enger, 2002). Oye and Rimstad (2001) reported a 3 log reduction in ISAV and VHSV titers after approximately 50 and 9 h, respectively, in sterilized seawater and freshwater. They also found that fish pen water enhanced viral survival compared to sterilized water. Skjold (2014) reported 3 log reductions in SA titer in 12 h. Garver et al. (2013) found that IHNV experienced threefold declines in viral titer <24 h, and that viral decay was much less in open aquaculture compared to closed aquaculture systems or sterile water. Kocan et al. (2001) reported a 50% loss of viral activity in VHSV when inoculated into natural seawater. Hence, viral infectivity decay rates of enveloped RNA viruses may be similar to those of virioplankton in general. The high decay rates present in seawater (detailed above) and high dilution rates suggest coronaviruses may not persist for long periods in natural waters, which would help to minimize the risk of infecting any potential susceptible hosts in the marine environment that could act as animal reservoirs of the virus. However, it is important to note that surviving virions may potentially infect marine mammals, since cetaceans and terrestrial mammals share similar receptor binding domains on ACE2 (Luan et al., 2020; Nabi and Khan, 2020) which may be especially pronounced when such species occur near urban wastewater outfalls. Marine aerosols may also represent another mechanism of re-introduction to terrestrial habitats downstream of wastewater outfalls (Baylor et al., 1977).

## Summary

In summary, coronaviruses occur uncommonly in marine and freshwater ecosystems as free virions, but this could be because the true diversity in aquatic reservoirs is not well explored. There may be unrecognized coronaviruses infecting marine metazoa that are currently under-sampled relative to terrestrial counterparts. Introduced coronaviruses, such as SARS-CoV-2, may be present in coastal marine waters which are affected by sewage effluent, where they are subject to physical decay and loss of infectivity at rates similar to other aquatic viruses. Monitoring of SARS-CoV-2 in sewage outfalls into these habitats is recommended, since it may provide guidance to recreational users and fisheries to assess risk, especially when such viruses may be concentrated, e.g., by filter feeding organisms or by onshore winds. More study is needed to understand the natural diversity of coronaviruses in marine metazoa through broad viral surveys.

## Author Contributions

GM and IH wrote the manuscript. Both authors contributed to the article and approved the submitted version.

## Conflict of Interest

The authors declare that the research was conducted in the absence of any commercial or financial relationships that could be construed as a potential conflict of interest.
